# Automatic Assessment of Socioeconomic Impact on Cardiac Rehabilitation

**DOI:** 10.3390/ijerph10115266

**Published:** 2013-10-25

**Authors:** Mireia Calvo, Laia Subirats, Luigi Ceccaroni, José María Maroto, Carmen de Pablo, Felip Miralles

**Affiliations:** 1 Cardiac Electrophysiology Unit, Hospital Clínic Universitari de Barcelona, Villarroel, 170, Barcelona 08036, Spain; 2 Barcelona Digital Technology Centre, Roc Boronat, 117, 5th floor, Barcelona 08018, Spain; E-Mails: lceccaroni@bdigital.org (L.C.); fmiralles@bdigital.org (F.M.); 3 Universitat Autònoma de Barcelona, Campus UAB, Bellaterra 08193, Spain; 4 Cardiac Rehabilitation Unit, Hospital Universitario Ramón y Cajal, Madrid 28034, Spain; E-Mails: jmmmcp@yahoo.es (J.M.M.); cpablozar@telefonica.net (C.P.)

**Keywords:** cost-effectiveness analysis, healthcare policy, disability-adjusted life years, quality-adjusted life years, ICF, cardiac rehabilitation

## Abstract

Disability-Adjusted Life Years (DALYs) and Quality-Adjusted Life Years (QALYs), which capture life expectancy and quality of the remaining life-years, are applied in a new method to measure socioeconomic impacts related to health. A 7-step methodology estimating the impact of health interventions based on DALYs, QALYs and functioning changes is presented. It relates the latter (1) to the EQ-5D-5L questionnaire (2) to automatically calculate the health status before and after the intervention (3). This change of status is represented as a change in quality of life when calculating QALYs gained due to the intervention (4). In order to make an economic assessment, QALYs gained are converted to DALYs averted (5). Then, by inferring the cost/DALY from the cost associated to the disability in terms of DALYs lost (6) and taking into account the cost of the action, cost savings due to the intervention are calculated (7) as an objective measure of socioeconomic impact. The methodology is implemented in Java. Cases within the framework of cardiac rehabilitation processes are analyzed and the calculations are based on 200 patients who underwent different cardiac-rehabilitation processes. Results show that these interventions result, on average, in a gain in QALYs of 0.6 and a cost savings of 8,000 €.

## 1. Introduction

The research described in this paper aims to provide a method to automatically assess, from existing *Medical Health Records* (MHRs), the socioeconomic impact of rehabilitation in a specific geographical area, considering, as a case study, the impact in Spain of cardiac rehabilitation. Cardiac rehabilitation attempts to reduce the levels of morbidity and impairment mainly caused by ischemic heart disease, the decrease in blood supply of the heart muscle usually due to coronary artery disease, which may result in angina pectoris, *Acute Myocardial Infarction* (AMI) or heart failure. Its role in improving the health of patients with ischemic heart disease has been recognized by the American Heart Association [1], the European Society of Cardiology [2] and the Spanish Society of Cardiology [3], among others. Cardiac rehabilitation is a multidisciplinary intervention offered to patients and their caretakers, providing a set of methods and instruments that attend to the multiple needs of the patient.

This article is also aimed to validate the cost-effectiveness of rehabilitation, illustrating the gains obtained in terms of quality of life and, ultimately, of cost savings related to health recovery. Indicators of health outcomes combining mortality and morbidity can provide qualitative and quantitative measurements of these gains related to health interventions, and can be used to provide decision support to policymakers in order to enhance healthcare management. In the case of cardiac rehabilitation, validation of cost-effectiveness is crucial to encourage its usage; especially in patients that have suffered from AMI, since in most European countries less than 50% of them undergo rehabilitation [4].

Ischemic cardiomyopathy is the leading cause of death in the World, accounting for 12% of total deaths, and the fourth leading cause of burden of disease, according to the World Health Organization (WHO) [5]. In Spain, as reported by the National Institute of Statistics (*Instituto Nacional de Estadística* or INE in Spanish,), ischemic cardiomyopathy is the main cause of mortality and one of the most common causes of morbidity and loss of quality of life. Patients may experience aggravating disability with increasing burden on their careers. They are also heavy users of healthcare and social-service resources, with an estimation of €2 billion per year being spent in Spain because of this disease [6].

*Disability-adjusted life years* (DALYs) and *Quality-adjusted life years* (QALYs), which capture life expectancy and quality of the remaining life-years, can be used in methods to measure socioeconomic impacts related to health, while *impairment* is increasingly being measured according to the *international classification of functioning, disability and health* (ICF) (see below for more details on these concepts). Several studies (e.g., [7,8]) have demonstrated a positive impact on quality of life in patients who have completed cardiac rehabilitation activities; and estimations of the costs involved in providing this kind of rehabilitation have been published [9]. However, we are not aware of any study that has directly calculated from MHRs data the overall socioeconomic impact of adding cardiac rehabilitation to the standard care of patients having suffered from AMI. Furthermore, we ignore the existence of any methodology which applies standard indicators to provide decision support to healthcare policy makers. Thus, the aim of this article is to suggest a novel methodology to automatically assess, from existing MHRs, the costs and benefits resulting from different cardiac rehabilitation processes.

### 1.1. Disability-Adjusted Life Years

DALYs are defined as the sum of the current value of future years of lifetime lost through premature mortality, and the current value of years of future lifetime adjusted for the average severity (frequency and intensity) of any mental or physical disability caused by a disease or injury [10]. They are therefore composed of two addends: (1) *years of life lost due to premature death* (YLL) and (2) *years lived with disability* (YLD). The second component is calculated as the discounted current value of years lived in a condition of impairment multiplied by a disability or severity weight for that condition, assigned in a scale of 0 (representing perfect health) to 1 (representing death). These weights are based on the outcomes of the *Global Burden of Disease Study* [5], in which different disability weights were allocated to each disease and impairment condition. Weights closer to 1 indicate that a year spent in that situation is perceived as closer to death than to a status of perfect health. Thus, being DALYs the number of perfectly healthy years of life lost, the severity of a disease can be measured taking into account the number of lost DALYs it causes. They are commonly used in cost-effectiveness analysis since, knowing the cost that losing DALYs represents for the society (cost/DALY), the economic savings due to DALYs averted by a health intervention can be obtained. However, DALYs do not consider the changes in people’s functional status or well-being if they receive rehabilitation services, assistive devices, accommodations, or live in a society that has become more open and accessible to individuals with functional limitations. They only reflect the presence of a medical condition without involving an empirical assessment of the functional or activity limitations actually experienced by those affected by an injury or disease [11]. According to recommendations found in the literature [12,13], an indicator based on ICF values (see below) is more consistent in reflecting the health recovery of patients whose medical-condition classification does not change.

### 1.2. Classification of Functioning, Disability and Health

The ICF belongs to a family of international classifications developed by the WHO. Its aim is to provide a unified and standard language and framework for the description of health and health-related status; it defines components of health and some health-related components of well-being. According to the WHO, disability derives from the interaction between functional limitations and an unaccommodating environment. People are not described as having a disability based upon a medical condition, but are rather described according to a detailed representation of their functioning, which uses these main classes: body functions, body structures, activities, participation and environmental factors. *Body Functions* is the domain most closely related to a medical model as it refers to the physiological and psychological functions of body systems. *Body Structures* are defined by the ICF as anatomic parts of the body such as organs, limbs and their components. *Activities* refer to a wide range of deliberate actions performed by an individual. They are actions undertaken in order to accomplish a task, such as walking or climbing stairs. *Participation* refers to activities that are integral to economic and social life, such as attending school or holding a job. *Environmental factors* make up the physical, social and attitudinal environment in which people live. A given level of impairment in body function will not necessarily translate into activity or participation limitations if the environment accommodates a person’s functional condition. And environment refers not only to the physical environment, but the cultural and political environment as well. Disability comes from participation restrictions that result from the interaction of many factors [13]. Each main domain includes more detailed subclasses up to 4 levels, so a person’s health condition can be defined with different precision by an array of components of the ICF rated using the same 0–4 scale, representing the level of impairment, limitation, restriction or barrier encountered, expressed as: (4) complete; (3) severe; (2) moderate; (1) mild and (0) no problem. There are already some studies where these values are used to assess the evolution of a patient in the management of rehabilitation processes (see [14,15,16]). In this study, ICF values are compared before and after rehabilitation for those classes that are needed when calculating the quality of life through the EQ-5D-5L questionnaire.

### 1.3. EQ-5D-5L Questionnaire

The precursor of EQ-5D-5L was the EQ-5D questionnaire from the EuroQol Group, a standardized, non-disease-specific instrument for describing and valuing health [17]. Applicable to a wide range of health conditions and treatments, it provides a simple, descriptive profile and a single value for health status that can be used in clinical and economic evaluation of healthcare as well as in population-health surveys. EQ-5D-5L is a brief, self-administered, two-page questionnaire: the first page contains five items describing health status across five dimensions (D): mobility, self-care, usual activity, pain/discomfort and depression/anxiety; the second page has a visual, analogue rating scale in which the respondent marks an assessment of his/her overall health status. Each dimension is divided into a scale of five levels (L), resulting in a total of 3,125 (5^5^) different possibilities of well-being status. The responses to the items in the EQ-5D-5L can be translated into health status weights using a utility-weighted algorithm [18], which has been recommended for use in economic evaluation. In this study, ICF values are extrapolated to the EQ-5D-5L questionnaire to obtain the weighted health status of each patient before and after a health intervention, such as cardiac rehabilitation.

### 1.4. Quality-Adjusted Life Years

QALYs provide a common currency to assess the extent of benefits gained from a variety of interventions in terms of health-related quality of life and survival for the patient [19]. QALYs assign a specific weight according to the time spent on different health status. A year of perfect health is worth 1 and a year of less than perfect health is worth less than 1. Death is considered to be equivalent to 0; however, some health statuses may be considered worse than death and have negative scores. QALYs are a good indicator for health interventions since they reflect a weighted health status and can be related to changes in ICF values.

### 1.5. Cost-Effectiveness Analysis and Socioeconomic Impact

Indicators used when making decisions in public health policies [20] compare the cost/DALY among interventions. In this way, an intervention is not only assessed based on how many DALYs it averts, but also on the economic impact of averting these DALYs. However, since DALYs are not a good measure of health recovery after rehabilitation, DALYs averted should be related to QALYs gained, taking into account the age-weighting implicit in the former. In this way, in a health intervention in rehabilitation, it is possible not only to assess the money invested to gain QALYs (as in cost-utility analysis) or to compare costs and economic benefits (as in cost-benefit analysis), but also to assess the amount of money saved averting DALYs. This kind of analysis, called cost-effectiveness analysis, provides the most general representation of the economic impact of health interventions. For the specific case of cardiac rehabilitation, a general need exists for analyzing the overall socioeconomic impact of the standard care of patients having suffered from an AMI.

**Table 1:** State of the art in socioeconomic evaluations of cardiac rehabilitation after acute myocardial infarction.

Title	Author	Socioeconomic evaluation	Country	Source
Economic evaluation of cardiac rehabilitation soon after acute myocardial infarction	Oldridge *et al*. 1993 [21]	cost/QALY	USA	MHRs
Cost-effectiveness of cardiac rehabilitation after myocardial infarction	Ades *et al*. 1997 [22]	cost/YLS	USA	Published trials and studies
A short course of cardiac rehabilitation program is highly cost effective in improving long-term quality of life in patients with recent myocardial infarction or percutaneous coronary intervention	Yu *et al*. 2004 [23]	cost/QALY	China	MHRs (Study 36-Item Short-Form Health Survey (SF-36) and Symptoms Questionnaire)
Cost-effectiveness of rehabilitation after an acute coronary event: a randomized controlled trial	Briffa *et al*. 2005 [24]	cost/QALY	Australia	MHRs
Rehabilitation of elderly with coronary heart disease-Improvement in quality of life at a low cost	Sandström *et al*. 2005 [25]	cost/QALY	Sweden	MHRs (EuroQol and Time Trade Off)
Economic burden of cardiovascular diseases in the enlarged European Union	Leal *et al*. 2006 [6]	cost/DALY	European Union	National ministries and statistical institutes
Cardiac rehabilitation—a cost analysis	Levin *et al*. 2009 [26]	cost/readmissions	Sweden	MHRs

Table 1 summarizes the state of the art in socioeconomic evaluations in the domain of cardiac rehabilitation. In some cases cost-effectiveness is used in order to provide a general evaluation of cost savings related to cardiac rehabilitation; however, cost-effective measures are based on indicators such as DALYs or *years of life saved* (YLS), therefore they do not reflect gains in terms of quality of life, an outcome extremely relevant when estimating the impact in patients with functional limitation. Other evaluations are based on the assessment of the cost-utility (cost/QALY) ratio. The methodology presented in this paper proposes to calculate the cost-effectiveness ratio from QALYs gained after a health intervention. Moreover, it estimates the impact of rehabilitation using data from questionnaires currently used in clinical practice, and enables the automation of the calculation of DALYs and QALYs.

## 2. Experiments

Patients perform cardiac rehabilitation in three stages, namely: (1) hospital stay; (2) rehabilitation in the hospital 2–5 times a week; and (3) whole life [27]. In the third stage, patients are advised to continue their whole life with the physical and mental learning program, and to practice sports achieving a specific heart rate. Relaxation exercises should also be performed regularly especially if patients are subjected to great mental tension. The available data provide information about the patients’ health status before the hospital stay (or before the first stage), before rehabilitation (or before the second stage) and after finishing rehabilitation (or after the second stage).

### 2.1. Cases

Cases of patients who had a myocardial infarction differ in their diagnosis, rehabilitation procedure and duration, the age of the patient and, consequently, the outcome after rehabilitation (expressed in ICF values of the classes relevant to perform the calculation of QALYs). The following examples correspond to three real cases of patients who suffered an acute myocardial infarction and performed different types of cardiac rehabilitation processes (motor, psychological, sexual rehabilitation).

Albert is the (anonymized) name of a 50-year-old man from Spain who suffered from an acute myocardial infarction. This refers to the condition when blood supply to part of the heart is interrupted because of a blockage in coronary arteries. When the myocardium (heart muscles) does not get enough oxygen, some of its cells die or become permanently damaged (necrosis). Two weeks after his hospital discharge, Albert starts the second stage of his cardiac rehabilitation. His initial evaluation shows he has some risk factors, such as smoking and hypertension, and some problems with his functional capacity (measured in metabolic equivalent of task or MET). His left ventricular ejection fraction (LVEF) is normal, and he does not have problems with anxiety, depression, dyslipidemia, sedentary lifestyle, diabetes, alcohol, abdominal perimeter or body mass index. His therapist plans a personalized treatment for him including motor rehabilitation and, after 115 days of therapy, she makes a final evaluation in order to assess his progress, measured in QALYs gained, cost/DALY and the overall cost savings due to the rehabilitation program performed.

Juan is the (anonymized) name of a 59-year-old man from Spain who suffered from an acute myocardial infarction. Two weeks after his hospital discharge, he starts the second stage of his cardiac rehabilitation. His initial evaluation shows that although his LVEF is normal, he has some risk factors, such as hypertension and dyslipidemia, and some problems with his functional capacity, anxiety and depression. His therapist plans a personalized therapy for him including motor and psychological rehabilitation and, after 157 days of rehabilitation, she makes a final evaluation in order to assess his progress, based on QALYs gained, cost/DALY and the overall cost savings due to the motor and psychological rehabilitation completed.

Pedro is the (anonymized) name of a 77-year-old man from Spain who suffered from a dilated cardiomyopathy. He also suffered from an ischemic cardiopathy with acute coronary syndrome without ST elevation and with incomplete surgical revascularization. Two weeks after his last hospital discharge, Pedro starts the second stage of his cardiac rehabilitation. His initial evaluation shows his LVEF is normal, but it also shows that he has some risk factors, such as dyslipidemia and problems with his functional capacity, erectile dysfunction, anxiety and depression. His therapist plans a personalized therapy for him which includes motor and sexual rehabilitation during 123 days. Finally, after the treatment, she would like to make a final evaluation in order to assess QALYs gained, cost/DALY and the overall cost savings due to the motor and sexual rehabilitation performed.

Motor rehabilitation refers to physical training consisting of walking three times a week on a treadmill placed at the hospital gym. Exercise intensity is based on the maintenance of the heart rate, increasing progressively the distance or endurance of the session so as to reach the goal of walking 6 km per day or walking for an hour, maintaining the targeted heart rhythm. Psychological rehabilitation begins with the evaluation of the patient through an interview in order to assess his/her anxiety, depression and personality. Based on the results obtained, the patient receives individualized treatment by psychologists and, if necessary, the psychiatrist of the unit. Sexual rehabilitation refers to the treatment of erectile dysfunction through drugs such as phosphodiesterase-5 inhibitors (sildenafil, sold as Viagra^®^, vardenafil, sold under the name of Levitra^®^, and tadalafil, sold under the name of Cialis^®^).

**Figure 1:**
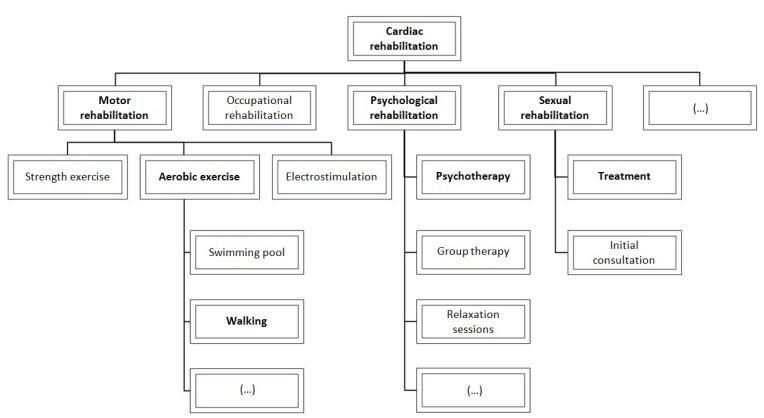
Cardiac rehabilitation’s map of processes.

Figure 1 represents the map of processes performed in cardiac rehabilitation programs at the Hospital Universitario Ramón y Cajal, highlighting in bold those processes completed by the three exemplifying patients.

The present study took into account a population of 200 patients. Data about the above three sample cases are summarized in Table 2, Table 3 and Table 4. ICF categories and values of Table 2, Table 3 and Table 4 are obtained from translating functional capacity in the exercise testing, occupational status and *Beck Depression Inventory* (BDI) following the methodology of Cieza *et al*. [28]. This approach consists of two basic steps: (1) standardize the item to one or several ICF categories and (2) normalize the value of the item for each ICF category, to a 0–4 scale. Mapping of questionnaires into ICF is shown in Table 5, where ICF values of deficiency are represented as (4) complete; (3) severe; (2) moderate; (1) mild and (0) no deficiency. The following Table 2, Table 3 and Table 4 show the values obtained in the functional capacity of the exercise testing, the BDI and the occupational status of the three exemplifying cases. The translation of the test scores into the ICF values is expressed in Table 5. The exercise testing assesses the patient’s ability to tolerate increased physical stress. It may be used for diagnostic, prognostic, and therapeutic applications, with or without addition of radionuclide or echocardiography assessment. After the exercise testing, patients can be classified according to the *Cardiac Functional Classification* [29] depending on whether they performed: 7 METs or greater (class I), between 5 and 6 METs (class II), 2 METs or greater but less than 5 METs (class III) and less than 2 METs (class IV). Functional capacity item is encoded into two ICF categories: mobility and self-care. The scores of classes I, II, III and IV are normalized (following the methodology of Cieza *et al*. [28]) into (4) complete; (3) moderate; (1) mild and (0) no difficulty in the ICF scale, respectively. Taking into account that self-care requires at least 2 METs [29], score of class IV is normalized into 4 (complete difficulty) and other scores are normalized into 0 (no difficulty). The BDI [30] is a questionnaire consisting of twenty-one queries about how the subject has been feeling in the last week in order to assess the emotional functions of the patient in the ICF scale. Each question has a set of four possible answer choices, ranging in intensity, assigning a value of 0 to 3 for each answer. The obtained score can be compared to a key to determine the depression’s severity of the patient meaning: (0–9) minimal, (10–18) mild, (19–29) moderate and (30–63) severe depression. The occupational status has two possible values: employed and unemployed, being translated into 0 (no difficulty) or 4 (complete difficulty) in the ICF scale.

In order to compare the outcomes of performing different types of rehabilitation, the study has been segmented in seven cohorts, in which *QALYs gained* and *cost saving* have been calculated:

(a) all patients (200 cases);
(b) patients who performed only motor rehabilitation (92 cases);
(c) patients who performed, at least, motor and psychological rehabilitation (24 cases);
(d) patients who performed only motor and psychological rehabilitation (14 cases);
(e) patients who performed, at least, motor and sexual rehabilitation (94 cases);
(f) patients who performed only motor and sexual rehabilitation (86 cases);
(g) patients who performed motor, psychological and sexual rehabilitation (10 cases).

**Table 2:** Albert’s case: 50-year-old man who participates in a **motor rehabilitation** process (walking) during 115 days.

	MHR value before/after rehabilitation	ICF indicator	ICF value before/after rehabilitation

Functional capacity of the exercise testing	6/9	Mobility (d4)	1/0
Functional capacity of the exercise testing	6/9	Self-care (d5)	0/0
Occupational status	Employed/Employed	Usual activities unspecified: Remunerative employment (d8509)	0/0
Beck Depression Inventory (BDI)	15/12	Pain/discomfort: Emotional functions (b152)	1/1
		Anxiety/depression: Emotional functions (b152)	1/1

**Table 3:** Juan’s case: 59-year-old man who participates in **motor and psychological rehabilitation** processes (walking and psychotherapy) during 157 days.

	MHR value before/after rehabilitation	ICF indicator	ICF value before/after rehabilitation

Functional capacity of the exercise testing	8/10	Mobility (d4)	0/0
Functional capacity of the exercise testing	8/10	Self-care (d5)	0/0
Occupational status	Employed/Employed	Remunerative employment (d8509)	0/0
Beck Depression Inventory (BDI)	21/6	Pain/discomfort: Emotional functions (b152)	2/0
		Anxiety/depression: Emotional functions (b152)	2/0

**Table 4:** Pedro’s case: 77-year-old man who participates in **motor and sexual rehabilitation** processes (walking and sexual treatment) during 123 days.

	MHR value before/after rehabilitation	ICF indicator	ICF value before/after rehabilitation

Functional capacity of the exercise testing	3/7	Mobility (d4)	3/0
Functional capacity of the exercise testing	3/7	Self-care (d5)	0/0
Occupational status	Unemployed/Unemployed	Remunerative employment (d8509)	4/4
Beck Depression Inventory (BDI)	19/6	Pain/discomfort: Emotional functions (b152)	2/0
		Anxiety/depression: Emotional functions (b152)	2/0

**Table 5:** Mapping of questionnaires into ICF.

Questionnaire	ICF Category	Questionnaire range/score (ICF score)
Functional capacity in the exercise testing	Mobility (d4)	≥7 (0), 5–6 (1), 2–4 (3), <2 (4)
Functional capacity in the exercise testing	Self-care (d5)	≥2 (0), <2 (4)
Occupational status	Usual activities unspecified: Remunerative employment (d8509)	Employed (0), unemployed (4)
Beck Depression Inventory (BDI)	Emotional functions (b152)	0–9 (0), 10–18 (1), 19–29 (2), 30–63 (4)

### 2.2. Methods

**Figure 2:**
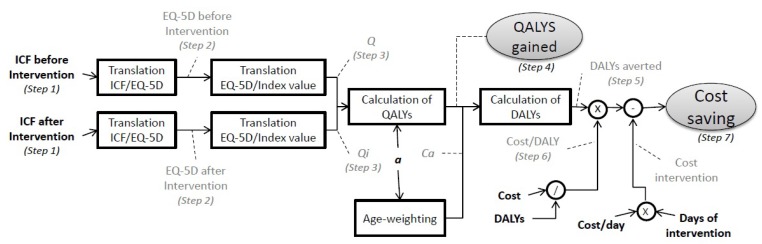
Block diagram of the methodology used.

A 7-step methodology is suggested to assess the impact of performing cardiac rehabilitation (or any other intervention which can be reflected in changes in ICF values). Figure 2 shows a block diagram in order to give a visual representation of the procedure. In dotted lines, the results of each step in the calculation can be found, to better keep track of the method used.

**Step (1)** The ICF values before and after rehabilitation for the ICF categories of Table 6 are identified. These categories are the ones relevant to perform the mapping into the EQ-5D-5L questionnaire. To compute EQ-5D-5L dimensions, if there are data of more than one ICF category, the average of their ICF categories will be performed following the methodology of Subirats *et al*. [31]. Table 6 shows the mapping of the questionnaire into ICF categories according to Geyh *et al*. [32]. The visual analogue rating scale, which refers to how good or bad the user feels his/her health is “today”, is also mapped into “emotional functions”. We can omit Step 1 if we already have the EQ-5D-5L values.

**Step (2)** The EQ-5D-5L values related to the ICF values found in Step 1 are identified. The 0–4 range of ICF values is mapped into the 1–5 range of the EQ-5D-5L questionnaire to obtain the weighted health status of each patient before and after cardiac rehabilitation.

**Step (3)** The values of the five top items in the EQ-5D-5L can be calculated using a utility-weighted algorithm [18], which has been recommended for use in economic evaluation. The index value calculator, downloadable from the EuroQol Group website, automatically calculates the health-status weights before and after rehabilitation from the EQ-5D-5L profiles.

**Step (4)** These health-status weights are used in QALYs as quality-weighted years of life. When used in order to calculate the gains in terms of life expectancy, quality-adjusted after rehabilitation, the number of QALYs gained is obtained as in Equation (1).

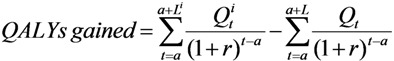
(1)
where *L* is the life expectancy for the subject at age *a*; *i* is the condition of a person after the rehabilitation process; *t* represents the individual years within the range of life expectancy; *r* is the discounting rate along years (*r* = 0.03); *Q* is the weight of the quality of life related to a year of life, calculated from the EQ-5D-5L values. Therefore, *L^i^* and *Q^i^* are the life expectancy and the weight of the quality of life after the rehabilitation process. We assume *L^i^* = *L*, which means the same years of life expectancy before and after rehabilitation, standardized to 80 years in males and 82.5 in females [19].

**Step (5)** In order to make a global economic assessment, we convert QALYs gained to DALYs averted, as in Equation (2). QALYs and DALYs are related by a conversion factor Ca, defined in Equation (3), where C = 0.1658 and β = 0.04, which takes into account the age-weighting implicit in DALYs, giving more weight to ages around 25 years [33].

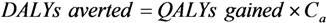
(2)

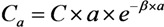
(3)

**Step (6)** The cost/DALY is identified, in order to give a global estimate of cost savings after having calculated the DALYs averted. The cost/DALY can be directly obtained from official documents or inferred from the cost associated to a disability in terms of DALYs lost, as in Equation (4).

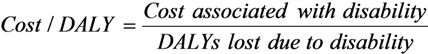
(4)
In our study we inferred this value.

**Step (7)** The overall cost savings due to a health intervention is calculated from the DALYs averted and the cost/DALY, taking into account the cost of the intervention, as in Equation (5).

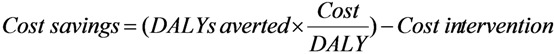
(5)

**Table 6:** Mapping of ICF categories into EQ-5D-5L dimensions.

EQ-5D-5L Dimension	ICF Category
Mobility	Mobility (d4), Walking (d450), Mobility, other specified (d498)
Self-care	Self-care (d5), Washing oneself (d510), Toileting (d530), Dressing (d540)
Usual Activities	Doing housework, unspecified (d6409), Family relationships, unspecified (d7609), Education, other specified and unspecified (d839), Remunerative employment, unspecified (d8509), Recreation and leisure, unspecified (d9209)
Pain/Discomfort	Emotional functions (b152), Sensation of pain (b280), Sensation of pain, other specified and unspecified (b289)
Anxiety/Depression	Emotional functions (b152)

### 2.3. Implementation

All steps except for steps 1 and 2 are implemented in a Java library, because if inputs are ICF indicators, they can be easily reused in other domains. The source code of the Java library and Javadoc documentation are freely available at [34]. The library has as inputs: *ICF indicators before and after intervention, age, cost of the disease, DALYs lost due to the disease* and *cost of the intervention*. Its outputs are: *QALYs gained* and *cost savings*.

## 3. Results

The assessment of the methodology is performed from patients’ data and the literature about DALYs and QALYs. Following steps 1–3 described in previous section, ICF values are mapped into the EQ-5D-5L questionnaire and, subsequently, into health status weights (see Table 7). The QALYs gained in each of the three cases described in Section 2.1, following Step 4, are:

*Motor rehabilitation’s case: QALYs gained* = 1.8

*Motor and psychological rehabilitation’s case: QALYs gained* = 3

*Motor and sexual rehabilitation’s case: QALYs gained* = 1.1

Beyond this comparison among interventions, in order to make a global economic assessment of the cases studied, we converted QALYs gained to DALYs averted (Step 5):

*Albert’s case: DALYs averted* = *QALYs gained* × C_50_ = 2

*Juan’s case: DALYs averted* = *QALYs gained* × C_59_ = 3

*Pedro’s case: DALYs averted* = *QALYs gained* × C_77_ = 0.6

The National Health System of Spain and the INE state that *myocardial ischemia* implies the loss of 227,000 DALYs each year, the 48.0% of which (109,000 DALYs) are because of acute myocardial infarction. Its cost is estimated in Spain at 1,955 million € [6], which means a cost/DALY of 17,940 €/DALY (Step 7). 

**Table 7:** Mapping of ICF values into health status weights *Q* and *Q^i^*.

	Before rehabilitation		After rehabilitation	
	ICF values	EQ-5D-5L values	ICF values	EQ-5D-5L values
**Albert’s case: motor rehabilitation**				
Mobility	1	2	0	1
Self-care	0	1	0	1
Usual activities	0	1	0	1
Pain	1	2	1	2
Anxiety	1	2	1	2
	Weight *Q*:	0.8	Weight *Q^i^*:	0.9
**Juan’s case: motor and psychological rehabilitation**				
Mobility	0	1	0	1
Self-care	0	1	0	1
Usual activities	0	1	0	1
Pain	2	3	0	1
Anxiety	2	3	0	1
	Weight *Q*:	0.8	Weight *Q^i^*:	1.0
**Pedro’s case: motor and sexual rehabilitation**				
Mobility	3	4	0	1
Self-care	0	1	0	1
Usual activities	4	5	4	5
Pain	2	3	0	1
Anxiety	2	3	0	1
	Weight *Q*:	0.2	Weight *Q^i^*:	0.5

Cardiac rehabilitation cost is analyzed in recent reports [9,35,36]. We assume that in Spain the cost is similar to research outcomes based on UK data, which are the most-recent, available data in the European Union. Taking this aspect and the context of the patients under study into consideration, the overall costs of undertaking cardiac rehabilitation is 14 €/day. Patients finish the second stage of rehabilitation after 115, 157 and 123 days; which means a cost of 1,600, 2,000 and 1,700 €, respectively. We can calculate then the economic saving of the DALYs averted with rehabilitation (Step 7):

*Albert’s case*: 17,940 × 2 − 1,600 = 30,000 €

*Juan’s case*: 17,940 × 3 − 2,000 = 50,000 €

*Pedro’s case*: 17,940 × 0.6 − 1,700 = 10,000 €

Albert undertook a motor rehabilitation program consisting of walking. After the therapy, his exercise test shows an improvement of 3 METs in his functional capacity, translated into a decrease of 1 point (from 1 to 0) in the ICF scale related to mobility. Thanks to the rehabilitation program followed, Albert has experienced a significant improvement in his mobility function which represents a betterment in his quality of life, measured with a gain of 1.8 QALYs, an avoidance of 2 DALYs and a saving for the society of 30,000 €.

Juan not only had some problems with his functional capacity, but also suffered from depression and anxiety. After his rehabilitation program consisting of walking and psychological treatment, the BDI registers a great improvement of 15 points translated into a decrease of two points in the ICF scale related to emotional functions. The outcome of completely eliminating moderate pain/discomfort and anxiety/depression is measured in a betterment of quality of life of 3 QALYs gained, 3 DALYs averted and 50,000 € saved.

Pedro is a 77-year-old man who had some problems with his functional capacity, as well as erectile dysfunction. After performing motor and sexual rehabilitation, he has experienced an improvement in his mobility and emotional functions, translated into a decrease of three and two points, respectively, in each class of the ICF. This represents a betterment in quality of life of 1.1 QALYs gained, 0.6 DALYs averted (quality of the remaining life years at 77 are not worth as much as at 50 or 59) and, thus, 10,000 € saved.

Table 8 shows the results obtained for mean QALYs gained and mean cost savings, when calculated for the seven populations described in Section 2.1.

**Table 8:** QALYs gained and cost savings under different sorts of cardiac rehabilitation.

	QALYs gained	Cost saving
All patients (200 cases)	0.6	8,000 €
Only motor rehabilitation (92 cases)	0.5	9,000 €
At least motor and psychological rehabilitation (24 cases)	0.5	7,000 €
Only motor and psychological rehabilitation (14 cases)	0.2	−200 €
At least motor and sexual rehabilitation (94 cases)	0.6	9,000 €
Only motor and sexual rehabilitation (86 cases)	0.6	9,000 €
Motor, psychological and sexual rehabilitation (10 cases)	1.0	16,000 €

## 4. Discussion

Based on the results obtained, performing motor, psychological and sexual rehabilitation at the same time results in the highest gain in quality of life and the most saving in costs. On the other end of the spectrum, performing motor and *psychological* rehabilitation at the same time results in little improvement in quality of life, and implies a positive comprehensive socioeconomic cost. As far as the other groups are concerned, they do not significantly diverge from the results obtained taking into account all patients for whom, on average, cardiac rehabilitation results in a gain in QALYs of 0.6 and cost savings of 8,000 €.

Due to changes of little magnitude observed, on average, in the quality of life of patients who undergo motor and *psychological* rehabilitation only and at the same time, it is suggested to allocate special attention when deciding about the eligibility for this combination of cardiac-rehabilitation processes. In these cases, according to the results obtained, adding sexual rehabilitation should be considered, if it makes sense for the specific patient under treatment. Nonetheless, it has to be taken into account that these results are based on changes in values of categories of the ICF, a classification which describes the functioning status using only five values. A patient could experience a slight recovery, to which the ICF is not sensitive. This representation in five levels particularly affects results when accounting for *psychological* changes using as data source the BDI questionnaire, described in Table 5, which has 64 values.

These results show the importance of cardiac rehabilitation after acute myocardial infarction. Although other studies had already proved its value by calculating gains in quality of life and cost-utility ratios, those measures were only able to compare interventions that modify QALYs in the same domain. From cost-utility ratios, it can be concluded the most effective intervention based on the minimum cost needed to gain a QALY. Although this outcome gives an objective measure when comparing interventions, it must be applied to the same disease or dysfunction, since costs of treating different pathologies are in most cases not comparable.

This study provides a measure to more globally assess the cost savings of performing cardiac rehabilitation so it can be compared to any other intervention in the overall framework of healthcare. Moreover, the methodology proposed is also applicable to measure any kind of recovery that can be expressed as changes in ICF values before and after an intervention.

## 5. Conclusions

Indicators of health outcomes can provide information about the quality of life gained after an intervention such as cardiac rehabilitation. These indicators, together with information provided by governments about the cost of lost quality of life in population and the cost of intervention to revert this, can be used to assess the impact of cardiac rehabilitation in patients who have suffered, e.g., an acute myocardial infarction. We presented a novel methodology to perform this assessment and validated the benefits of rehabilitation in terms of enhancing quality of life and giving decision support to healthcare policymakers. This methodology, which can be used with data from existing medical health records, takes into account changes in people’s functional status, represented by ICF values, resulting from interventions that produce quality-of-life gains but do not change an underlying medical diagnosis.

In order to obtain an economic evaluation, QALYs were converted into DALYs and the cost/DALY indicator was calculated from the cost associated to a disability and the DALYs lost due to that disability. The proposed socioeconomic assessment, which was validated with data of 200 patients taking part in cardiac rehabilitation, showed that this health intervention offers, on average, a gain in QALYs of 0.6 and cost savings of 8,000 €. However, since the methodology presented is based on changes in the ICF, which describes functioning status using a scale of five levels, a patient could experience a slight recovery to which the ICF is not sensitive.

We are planning to extend the use of the proposed approach for: (1) automatic socioeconomic impact assessment of other sorts of rehabilitation processes, such as functional, cognitive or respiratory rehabilitation; (2) QALYs evolution in periodic, comprehensive and multidimensional evaluations of people with disabilities of neurological origin; and (3) QALYs evolution in telemonitoring systems of people with functional diversity in which brain-neural computer interfaces are used. Furthermore, the generalization of this methodology to other diseases and rehabilitation processes can be a valuable tool to provide information on and evaluate health policies.

## Supplementary Files

Supplementary File 1 Supplementary (RAR, 10982 KB)
